# Effect of CYP3A4 metabolism on sex differences in the pharmacokinetics and pharmacodynamics of zolpidem

**DOI:** 10.1038/s41598-021-98689-z

**Published:** 2021-09-27

**Authors:** Seonghae Yoon, Seongmee Jeong, Eben Jung, Ki Soon Kim, Inseung Jeon, Yujin Lee, Joo-Youn Cho, Woo-Yong Oh, Jae-Yong Chung

**Affiliations:** 1 Department of Clinical Pharmacology and Therapeutics, Clinical Trials Center, Seoul National University Bundang Hospital, 82, Gumi-ro 173beon-gil, Bundang-gu, Seongnam, 13620 Republic of Korea; 2 Clinical Research Division, National Institute of Food and Drug Safety Evaluation, Ministry of Food and Drug Safety, Cheongju, Republic of Korea; 3 Department of Clinical Pharmacology and Therapeutics, Seoul National University College of Medicine and Hospital, Seoul, Republic of Korea; 4 Department of Biomedical Sciences, Seoul National University College of Medicine, Seoul, Republic of Korea

**Keywords:** Health care, Medical research

## Abstract

To investigate pharmacokinetic and pharmacodynamic differences of zolpidem between males and females and their causes, including CYP3A4 activity. A single oral dose of zolpidem (10 mg) was administered to 15 male and 15 female healthy subjects. Blood samples were collected up to 12 h post-dose to determine plasma zolpidem concentrations. Pharmacokinetic parameters were obtained using non-compartmental analysis. Digit symbol substitution test, choice reaction time, and visual analog scale of sleepiness were used to evaluate pharmacodynamics. We measured CYP3A4 activity using 4β-hydroxycholesterol, an endogenous metabolite. Mean maximum plasma concentration and area under the plasma concentration–time curve were higher for females than for males (9.9% and 32.5%, respectively); other pharmacokinetic parameters showed no significant differences. Pharmacodynamic scores for females showed delayed recovery compared with that for males. CYP3A4 activity was higher in females than in males (*p* = 0.030). There was no serious adverse event, and adverse event incidence was not different between the sexes. Zolpidem exposure was about 30% higher in females than in males. Delayed pharmacodynamic score recovery in females could be related to higher zolpidem concentrations. Although apparent clearance was lower in females, systemic clearance might not be the cause of the different exposures to zolpidem.

## Introduction

Zolpidem is a non-benzodiazepine hypnotic agent used to treat patients with insomnia, a disorder characterized by difficulties with sleep initiation. According to recent studies, 3.8–4.8 million adults per year are prescribed zolpidem in the USA, and two-thirds of patients are female^1,2^. Zolpidem is also a recommended treatment for patients with insomnia in Korea, where it is currently the most widely used hypnotic agent. Similar to other countries, it has been found that sedative-hypnotics are more frequently prescribed to female patients than males^3^.

There are only few cases where drug labels recommend prescribing drug dosage regimens based on sex. Desmopressin is among those few cases, which is prescribed for treating nocturia, and have pharmacokinetic (PK) and pharmacodynamic (PD) differences, particularly in drug response^4,5^. Another example is suvorexant, which is a drug for insomnia: exposure of suvorexant is increased in obese to non-obese patients, and in women compared to men^6^. As such, these drugs may benefit from differing sex-based dosage regimens. In 2013, labeling information of zolpidem was changed in the USA; the initial recommended dose of women was reduced from 10 to 5 mg of immediate-release formulation and from 12.5 to 6.25 mg of controlled-release formulation, whereas men could be prescribed either dosage^7,8^. This decision was made due to concerns that women could have higher drug concentrations than men and, subsequently could be at increased risk of driving impairment^9^. However, other regulatory agencies, including the European Medical Agencies (European Union), Pharmaceuticals and Medical Devices Agency (Japan), and Ministry of Food and Drug Safety (Korea) have not changed the initial recommended dose until now. While different initial dose is recommended in different sex only in the USA, the initial dose of elderly is 5 mg in all other countries^10–12^.

Several studies have explored the PK and/or PD of zolpidem and compared its action between males and females^8,13–18^. Zolpidem exposure in females, represented as maximum drug concentration (C_max_) and area under the concentration–time curve (AUC), was consistently higher than in males in all studies. Even though the apparent clearance (CL/F) of zolpidem was higher in females than in males in most studies, the difference in CL/F was smaller when normalized to the body weight of subjects. Most studies used oral or sublingual routes and only CL/F was calculated, which can also be influenced by bioavailability (F). In more than half of the studies, the extent of elimination (i.e., the half-life (t_1/2_)) did not differ between males and females. However, the causes of the differences in these studies have not been clarified. Although some studies have evaluated sex differences in PD^8,13,15^, these differences were primarily owing to higher concentrations in females, and the differences in the concentration–effect relationship were not definite.

Zolpidem is extensively metabolized and < 1% of the dose is excreted unchanged in urine^19–21^. Zolpidem is metabolized mainly by cytochrome P450 (CYP) 3A4 (61%), CYP2C9 (22%), CYP1A2 (14%), and CYP2D6 (2.5%)^20^. CYP3A4 appears to have a greater expression in females than in males, as determined by mRNA and protein levels^22–24^ and its metabolic activity is also higher in females^24^. Therefore, we questioned the hypothesis that higher exposure to zolpidem in females is owing to its lower clearance (CL).

To date, no study has compared the PK and/or PD characteristics of zolpidem between Asian males and females. This study aimed to investigate whether there is any difference in zolpidem PK/PD between males and females and subsequently identify the factors that cause this difference. We also evaluated CYP3A4 activity using an endogenous biomarker and examined its correlation with the PK of zolpidem.

## Methods

### Study design and participants

This study was designed as an open-label, single-dose study. The study was performed in accordance with the Declaration of Helsinki and the Korean Good Clinical Practice guidelines and was registered on the research registration website of Clinical Research Information Service (http://cris.nih.go.kr, KCT0003934, date of registration: 2018-08-29) and approved by the institutional review board of Seoul National University Bundang Hospital (B-1802-453-002). All subjects provided written informed consent before participating in the study.

We planned to enroll 30 subjects (15 for each group) considering the exploratory nature of this study and previous studies’ sample size. The sample size of 30 has 80% power to show a 40% difference in a PK parameter with a coefficient of variation of 30% (alpha level of 0.05).

Healthy male and female subjects, aged between 20 and 45 years, were enrolled based on their medical history, vital signs, 12-lead electrocardiogram, clinical laboratory tests, and physical examination at the screening. Only non-smokers were enrolled, and intake of other concomitant drugs or food that could affect the metabolism of zolpidem was restricted during the study period. During the hospitalization period, only provided meals were consumed. Subjects were admitted on day-1 for baseline tests, and a single oral dose of zolpidem 10 mg was administered on day 1 in the morning. They were discharged after completing 12 h post-dose procedures. Safety and tolerability were assessed through vital signs, physical examinations, clinical laboratory tests, and adverse event (AE) monitoring.

### Quantification of zolpidem and PK analysis

Blood samples for PK analysis were collected using K2 EDTA tubes to determine plasma concentrations of zolpidem, at pre-dose and 0.25, 0.5, 0.75, 1, 1.5, 2, 3, 4, 6, 8, and 12 h post-dose. Tubes were centrifuged at 3000 rpm, 4 °C for 10 min, and the plasma was collected and stored at − 70 °C until use.

A fully validated quantification method using ultra-performance liquid chromatography (UPLC)-tandem mass spectrometry was used to determine zolpidem concentrations. Zolpidem tartrate and doxazosin mesylate (internal standard, IS) were provided by the Ministry of Food and Drug Safety in the Republic of Korea. Plasma samples were extracted by protein precipitation with acetonitrile. Chromatographic separation was performed at 35 °C on an ACQUITY UPLC I-Class UPLC (Waters Corporation, Milford, MA, USA) using an HSS T3 column (2.1 mm × 100 mm, 1.8 μm). The mobile phase consisted of a mixture of acetonitrile and 5 mM ammonium acetate (pH 3.5, adjusted by formic acid; 40:60 v/v). The flow rate was 0.2 mL min^−1^. The UPLC system was coupled to a mass spectrometer (LTQ Orbitrap Elite™, Thermo Scientific, Bremen, Germany) equipped with an electrospray interface operated in the positive ionization mode. The mass-to-charge transition was monitored for the quantification of zolpidem and IS. The value was *m/z* 308.17 → 235.12 and *m/z* 452.17 → 344.09, respectively. The method has been fully validated in terms of selectivity, matrix effect, carry-over, lower limit of quantification (LLOQ), linearity, accuracy, precision, recovery, and stability. The calibration curves were linear over the concentration range from 0.5 to 500 ng mL^-1^ with a correlation coefficient of r^2^ ≥ 0.99. The best linear fit and least-squares residual for the calibration curves were achieved by employing 1/x^2^ as a weighting factor. The LLOQ was 0.5 ng mL^−1^. Within-run and between-run accuracy and precision were 13% and 5%, respectively. Zolpidem tartrate and IS were recovered at 92.8% and 94.3%, respectively. The stock and working solutions were stable for 10 days. Plasma samples were stable for 4 h at room temperature, within the first three freeze–thaw cycles, for 24 h at 4 °C, and for 77 days at − 70 °C.

The PK parameters were calculated using non-compartmental analysis using Phoenix WinNonlin software version 8.1 (Certara, Princeton, NJ, USA). Data comparison between groups was conducted using the *t*-test in R version 3.5.1^25^.

### CYP3A activity analysis

To evaluate the CYP3A activity of subjects, we quantified an endogenous marker, 4β-hydroxycholesterol in plasma. To quantify 4β-hydroxycholesterol, we used a 7890B series gas chromatograph (Agilent Technologies, Santa Clara, CA, USA) coupled with a 7000B series triple quadrupole mass spectrometer (Agilent Technologies) (GC–MS/MS)^26^. For the quantification of 4β-hydroxycholesterol, 50 µL of each plasma sample was saponified and liquid–liquid extracted. The extracted samples were evaporated using a nitrogen evaporator at 37 °C and derivatized with a mixture of N-methyl-N-(trimethylsilyl)trifluoroacetamide, ammonium iodide, and 1,4-dithioerythritol (200:2:1, v/w/w) at 60 °C for 20 min. Finally, 3 µL of each sample was injected into the GC–MS/MS. The concentrations of 4β-hydroxycholesterol in the plasma were determined from the calibration curve. The determined r^2^ was > 0.99, and the concentrations are expressed as ng mL^−1^. The between-run accuracy in plasma samples ranged from 97.52 to 106.8%. The between-run precision (coefficient of variation, CV) ranged from 2.22 to 5.34%, respectively. All quality control concentrations were consistent with the calculated amounts; the acceptable accuracy and CV were within at least 15% in all analytes.

### PD analysis

We conducted the following PD tests to evaluate the effect of zolpidem: digit symbol substitution test (DSST)^27^, choice reaction time (CRT) test^28,29^, and self-rating visual analog scale (VAS) of sleepiness with horizontal numerical rating^30^. Three PD tests were conducted at planned time points. On day-1, baseline tests were conducted at − 23, − 22, − 20, and − 12 h prior to dosing. On day 1, PD tests were conducted at 0.5, 1, 2, 3, 4, 6, 8, 12 h post-dose. DSST is a commonly used neuropsychological test in which the subject writes down the matching symbol for each digit and the number of correct symbols within 90 s is measured. The CRT test can assess general alertness and motor speed in which multiple stimuli, such as displayed arrows or cross and response time, are measured. Each subject rated their subjective sleepiness using VAS on a scale from 0 to 100^30^. PD test results were presented with baseline corrected values because of the difference in baseline values between males and females.

### Statistical analysis

All statistical analyses were performed using R version 3.5.1^22^. All demographic, PK, and PD data were summarized using descriptive statistics (mean [SD] or median [range]). Geometric mean ratios (GMR) and their 95% confidence intervals (CI) of major PK parameters were calculated. PD parameters were corrected to baseline levels and we performed two-way repeated measures analysis of variance (RM-ANOVA) to evaluate PD changes over time. Demographic data, PK, and PD parameters were compared between groups using the *t*-test or Wilcoxon-rank sum test.

### Ethics approval

This study was approved by the institutional review board of Seoul National University Bundang Hospital (B-1802-453-002).

### Consent to participate

All subjects provided written informed consent prior to participating in the study.

## Results

### Study participants and demographic characteristics

A total of 30 subjects (15 male and 15 female) were enrolled in and completed the study according to the study protocol. There was no difference in age between males and females, however, height and body weight were higher in males (Table 1).

**Table 1:** Demographic characteristics.

	Male (n = 15)	Female (n = 15)	*p* value*
Age (years)	30.2 ± 5.9	29.9 ± 6.2	0.739
Height (cm)	173.5 ± 5.5	159.9 ± 5.6	< 0.001
Weight (kg)	73.1 ± 7.6	54.7 ± 3.9	< 0.001
BMI (kg m^−2^)	24.3 ± 2.6	21.4 ± 1.4	0.003
4β-OH cholesterol (ng mL^−1^)	27.0 ± 9.9	38.7 ± 15.7	0.030

All values are presented as arithmetic mean ± standard deviation.

*p value: calculated by Wilcoxon rank-sum test.

*BMI* body mass index.

### PK and relationship with sex

**Figure 1:**
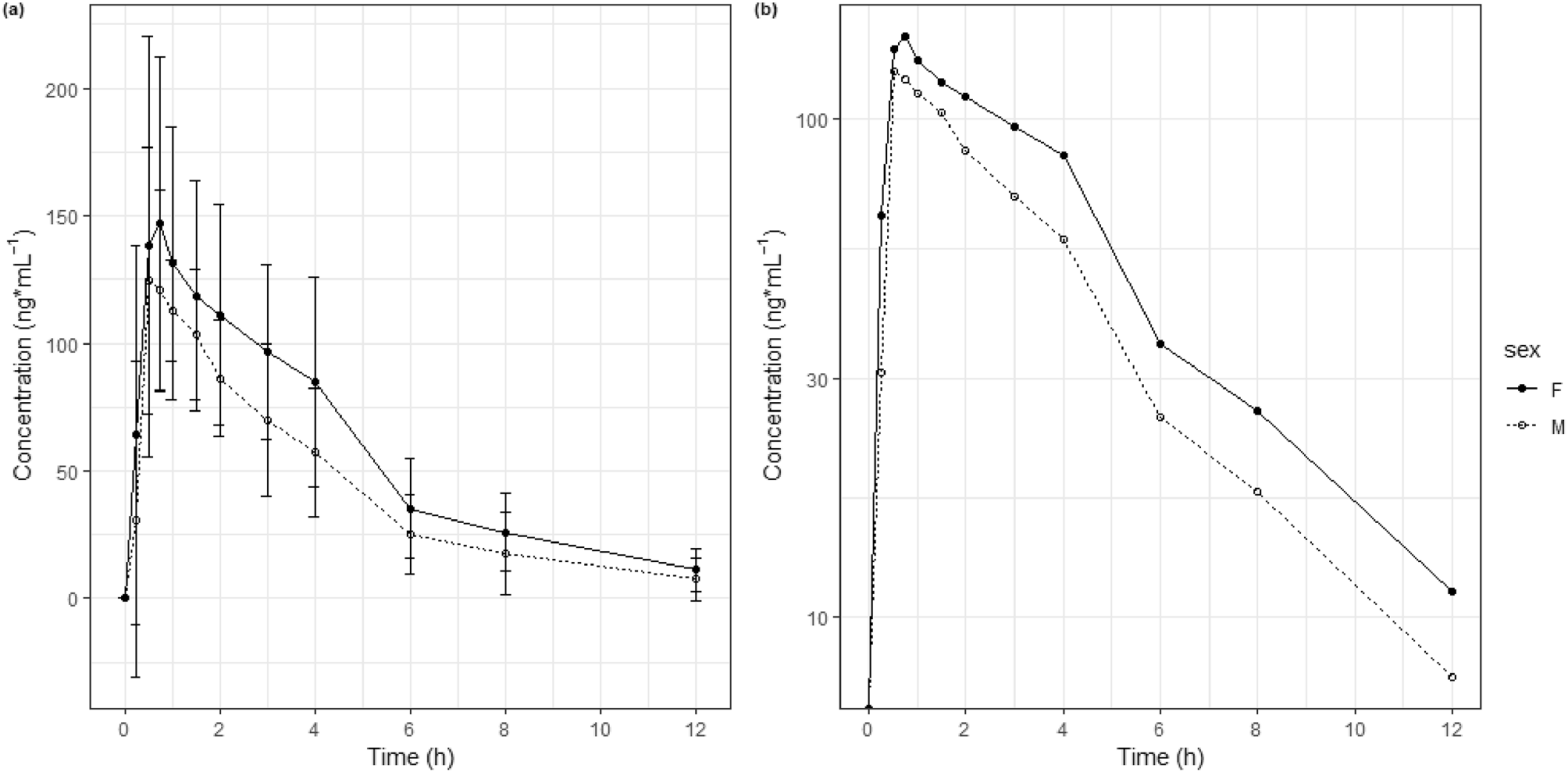
Mean plasma concentration–time profiles of zolpidem. (**a**) Linear scale, (**b**) log-scale. Female: Closed circle-solid line; Male: Open circle-dotted line, Bars represent standard deviation.

Zolpidem was absorbed rapidly, reaching maximum concentrations within 1 h in most subjects (Fig. 1). The median time to reach C_max_ (T_max_) was 0.9 and 1.1 h for males and females, respectively (Table 2). Mean plasma levels were higher in female subjects than in male subjects at all time points. The mean C_max_, AUC_0-12 h_, and AUC_inf_ were 10.9%, 31.0%, and 32.5% higher in women than in men (Table 3). It seems differences were not significant considering p values. However, it can be interpreted AUC_0-12 h_ and AUC_inf_ were higher in females considering GMR did not include 1.000. The t_1/2_ was longer in females than in males, but there was no statistical difference. The weight normalized CL/F and volume of distribution were also not significantly different between the sexes. After 8 h of zolpidem administration, most subjects had zolpidem concentrations of less than 50 ng mL^−1^, except one male (69.0 ng mL^−1^) and one female subject (60.0 ng mL^−1^).

**Table 2:** PK parameters of zolpidem after single oral administration.

	Male (n = 15)	Female (n = 15)	*p* value
T_max_ (h)	0.5 (0.25–1.50)	0.75 (0.25–4.0)	0.604^‡^
C_max_ (ng mL^−1^)	154.0 ± 44.5	172.9 ± 59.9	0.335^†^
C_8h_ (ng mL^−1^)	17.9 ± 16.2	25.9 ± 15.2	0.061^‡^
C_12h_ (ng mL^−1^)	7.6 ± 8.5	10.5 ± 8.5	0.116^‡^
AUC_0-12 h_ (h ng mL^−1^)	502.8 ± 197.3	671.2 ± 264.6	0.054^†^
AUC_inf_ (h ng mL^−1^)	544.5 ± 254.8	727.2 ± 310.6	0.084^†^
t_1/2_ (h)	2.9 ± 1.0	3.1 ± 0.7	0.106^‡^
CL/F (L h^−1^)	21.4 ± 7.8	16.6 ± 8.2	0.117^†^
CL/F (L h^−1^ kg^−1^)*	0.3 ± 0.13	0.3 ± 0.16	0.806^‡^
Vd/F (L)	80.3 ± 17.4	68.8 ± 19.6	0.138^†^
Vd/F (L kg^−1^)*	1.07 ± 0.2	1.25 ± 0.41	0.436^†^

*Weight normalized value; ^†^*t*-test; ^‡^Wilcoxon rank-sum test.

All values are presented as arithmetic mean ± standard deviation except for T_max_, for which median [minimum – maximum] is presented.

*C*_*8h*_ concentration at 8 h post-dose, *C*_*12h*_ concentration at 12 h post-dose, *C*_*max*_ maximum plasma concentration, *T*_*max*_ time to reach C_max_, *AUC*_*0-12 h*_ area under the concentration curve from 0 to 12 h, *AUC*_*inf*_ area under the concentration curve from 0 to infinity, *t*_*1/2*_ half-life, *Vd/F* apparent volume of distribution, *CL/F* apparent clearance.

**Table 3:** Geometric mean ratio and 90% confidence interval of pharmacokinetic parameters of zolpidem between sexes.

	Male (n = 15)	Female (n = 15)	Geometric mean ratio* (90% CI)
C_max_ (ng mL^−1^)	148.2	162.8	1.0986 (0.8950–1.3486)
AUC_0-12 h_ (h ng mL^−1^)	472.8	619.5	1.3101 (1.0258–1.6733)
AUC_inf_ (h ng mL^−1^)	501.6	664.4	1.3246 (1.0151–1.7283)

*Geometric mean ratio of females to males.

*C*_*max*_ maximum plasma concentration, *AUC*_*0-12 h*_ area under the concentration curve from 0 to 12 h, *AUC*_*inf*_ area under the concentration curve from 0 to infinity.

### PD and relationship with sex

**Figure 2:**
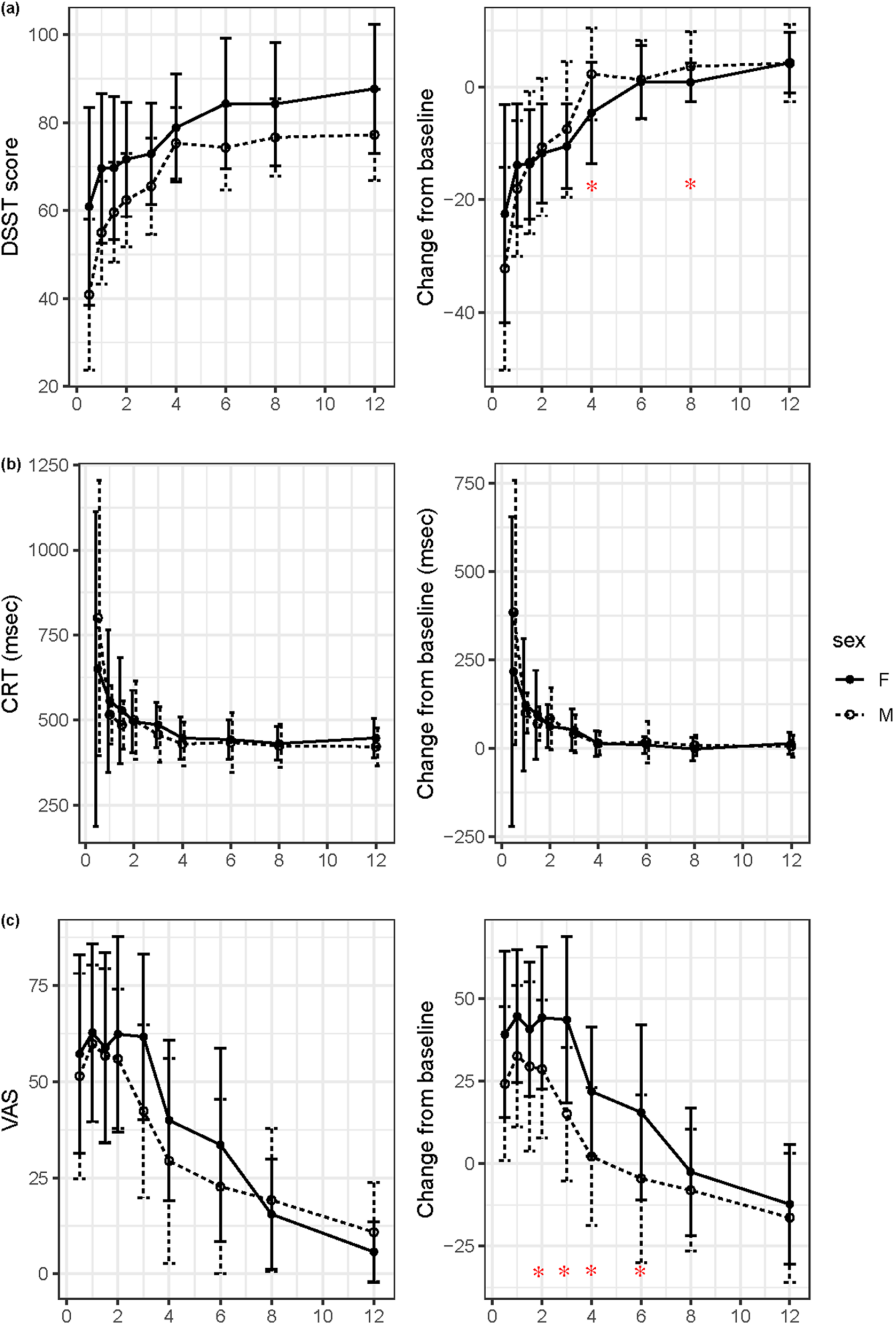
Pharmacodynamic parameters and their changes from baseline after single oral administration of zolpidem. (**a**) Digit symbol substitution test (DSST) score, (**b**) choice reaction time (CRT) test, and (**c**) self-rating visual analog scale (VAS) of sleepiness. The left panel shows the PD test result itself, and the right panel shows the baseline corrected result. Bars represent standard deviations and asterisks (*) means that the mean difference is statistically significant between males and females (*p* < 0.05). Digit symbol substitution test (DSST)^24^, choice reaction time (CRT) test^25,26^, and self-rating visual analog scale (VAS) of sleepiness^27^.

Among the three PD parameters, DSST scores decreased after zolpidem intake owing to decreased alertness (Fig. 2). The reaction time of CRT and the VAS score increased after zolpidem intake. The changes were the largest at 0.5 h except for the VAS, for which the largest changes occurred between 0.5 and 3 h because some subjects had a delayed response in VAS. The time of the greatest change in DSST score and CRT coincided with the T_max_ of zolpidem, which implies that zolpidem had an immediate effect on alertness and psychomotor function. However, the time to the largest change in subjective sleepiness measured by VAS was slightly delayed compared with the T_max_ of zolpidem.

Even though the pattern of PD change over time was similar between males and females, the recovery time to baseline level was longer in females, especially in DSST and VAS. Although the DSST score in the male group recovered 2 h after zolpidem intake, it took 4 h in the female group: it was judged to be recovered when there was no statistical difference from the baseline values. The DSST score change from the baseline at 4 and 6 h was significantly different between males and females. The CRT change was immediate and there was no difference between sexes. Two-way RM-ANOVA showed that the VAS score change difference between sexes was statistically significant (*p* = 0.0183). Although the VAS score in males recovered back to baseline levels in 3 h, it took 6 h to recover in females. The VAS score change from baseline was significantly different at 2, 3, 4, and 6 h between males and females.

### PK-PD relationship

**Figure 3:**
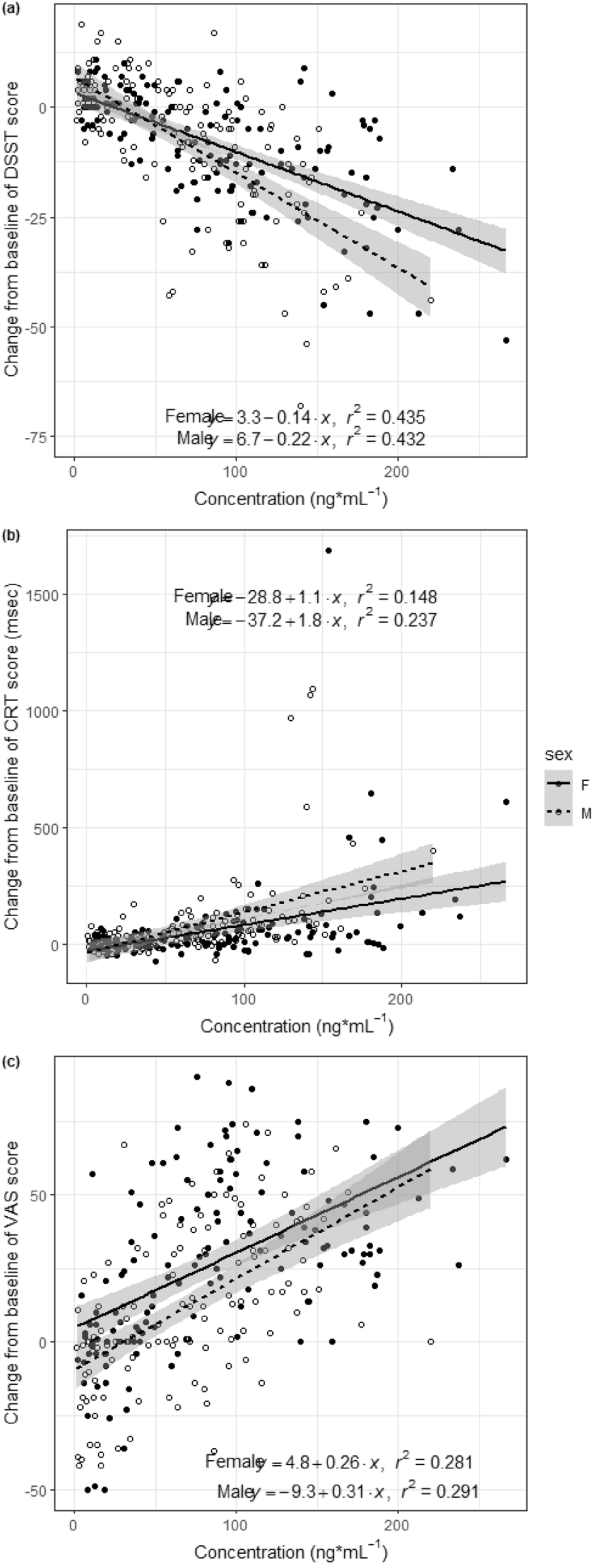
Pharmacokinetics–pharmacodynamics relationship of zolpidem. The relationship between zolpidem concentration and (**a**) digit symbol substitution test (DSST) score changes, (**b**) choice reaction time (CRT) changes, and (**c**) self-rating visual analog scale (VAS) of sleepiness changes. The y-axis represents changes in each pharmacodynamic parameter.

The relationship between drug concentration and PD parameter changes was also analyzed. Whereas the DSST score change had a negative linear relationship with the zolpidem concentration, the CRT score and VAS score change had a positive linear relationship with the zolpidem concentration (Fig. 3). To evaluate sex differences in the PK-PD relationship, the slope of the linear regression line was compared. The slope of the regression line of the DSST score change was significantly steeper in males than in females (*p* = 0.001). The slope of the regression line of the CRT change and VAS score change did not differ between the sexes (*p* = 0.076 and 0.332, respectively).

### PK-CYP3A activity relationship

**Figure 4:**
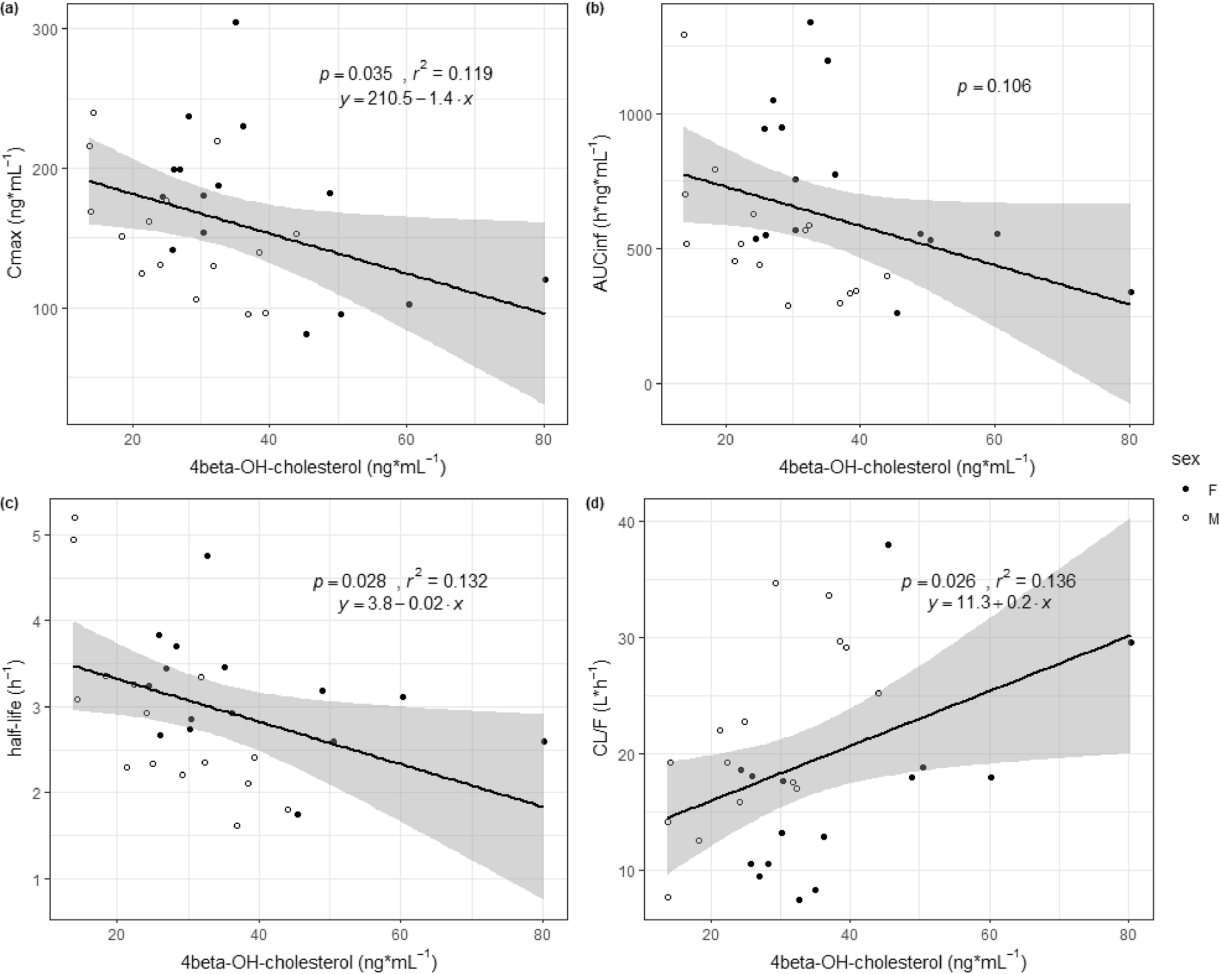
Relationship between pharmacokinetic parameters and CYP3A activity. The relationship between CYP3A activity (4beta-OH cholesterol) and (**a**) C_max_, (**b**) AUC_inf_, (**c**) half-life, and (**d**) CL/F.

The mean levels of 4β-OH cholesterol (SD) were 27.0 (9.9) and 38.7 (15.7) for males and females, respectively (*p* = 0.030). Females had significantly higher CYP3A activity than males, represented by 4β-OH cholesterol levels (Table 1). We analyzed the relationships between PK parameters and CYP3A activity represented by the 4β-OH cholesterol level (Fig. 4). Among various PK parameters, the C_max_, t_1/2_, and CL/F had a significant relationship with CYP3A activity. The higher the CYP3A activity, the lower the C_max_ and AUC. The higher the CYP3A activity, the shorter the t_1/2_. CL/F also had a positive relationship with CYP3A activity.

### Safety assessments

A single dose of zolpidem was safe and well-tolerated. Six cases of AEs (five of nausea and one of dizziness) were reported in six subjects (two males and four females), and there was no difference in the incidence of AEs between the sexes. All AEs were mild in severity, and no serious AEs were reported.

## Discussion

Zolpidem is a widely used hypnotic agent and has different PK profiles between males and females. We investigated PK and PK-PD relationship differences between males and females and the cause of these differences.

Several studies have analyzed the PK and/or PD characteristics of zolpidem and compared these characteristics between males and females^8,13–18^. We summarized the design information and PK analysis results of those studies (Supplementary Table 1, 2). Most studies have shown that exposure to zolpidem is higher in females than in males. The mean values of C_max_, AUC_0-12 h_, and AUC_inf_ were also higher in females in our study, but the difference was not statistically significant. The CL/F of zolpidem is significantly lower in females than in males in most studies. When normalized to body weight, this statistical significance disappears, except for a study conducted on Chinese patients^17^. The fact that weight-normalized CL/F is not different between sexes implies that body weight contributes somewhat to sex differences in CL/F. Even though the CL/F was 22% lower in females in our study, it was not statistically significant. Weight-normalized CL/F was also not statistically significant.

Zolpidem is extensively metabolized and CYP3A4 contributes to approximately 60% of zolpidem metabolism^16^. Although the PK parameter analysis results are similar among studies, we evaluated CYP3A4 metabolic activity represented by the endogenous biomarker 4β-OH cholesterol level, which is an endogenous biomarker that is formed by the CYP3A4 enzyme and could be used as a biomarker to evaluate CYP3A4 enzyme activity^31–35^. CYP3A4 metabolic activity is higher in females^24^. Even though sex differences in CYP3A4 activity seem evident, differences in CYP2C9 and CYP2D6 metabolic activity are not clear, and CYP1A2 activity is higher in males than in females^22,36^. The baseline CYP3A4 activity was higher in females in our study, as in previous studies^24,34^. CYP3A4 activity and the terminal t_1/2_ of zolpidem were also negatively correlated (Fig. 4). In other words, the greater the CYP3A4 activity, the faster the drug was cleared from the body. However, the t_1/2_ was not different between males and females, possibly because multiple metabolic enzymes are involved in the metabolism of zolpidem, not only CYP3A4.

The CL/F is calculated using the following equation: CL/F = dose/AUC_inf_. Increased exposure to drugs could be explained by increased F or decreased CL. It is difficult to evaluate F and CL separately in studies using the oral route of administration. Even though exposure to zolpidem is higher and CL/F is lower in females than in males, it does not necessarily mean lower CL in females. Considering that the terminal t_1/2_ is not statistically different between the sexes in more than half of the studies we reviewed, including our study, a systemic clearance difference might not be the major cause of increased exposure in females. The lower body weight of females could be one explanation for the increased exposure. It is difficult to calculate the absolute F without PK results of intravenous zolpidem administration. However, considering CL/F is lower and CL is similar or higher in females, the F could be higher in females than in males. The CL/F ratio was lower in females, but it is not accurate to state that the clearance (or metabolism) of zolpidem is lower in females. The apparent volume of distribution (Vd/F) was a little bit lower in females but it was not statistically different. The lower Vd/F leads to a higher C_max_ in females. Weight-normalized Vd/F is higher in females, which is plausible considering zolpidem is slightly lipophilic^19^.

We evaluated three PD parameters in this study: DSST, CRT, and self-rating VAS of sleepiness. The effect of zolpidem was immediate because the peak response time of PD tests was similar to T_max._ These rapid onsets of PD effects were consistent with the previous study^37^. To evaluate sex difference and PD change over time, we performed two-way RM-ANOVA. VAS score change was significantly different between sexes (*p* = 0.0183). Although CRT recovery was similar between sexes, DSST and VAS score recovery was delayed in females. The DSST score recovered to baseline levels 2 h post-dose in males and 4 h in females, whereas the VAS score recovered to baseline 3 h post-dose in males and 6 h in females. Other studies evaluating PD markers showed similar results; the effects on self-rated sedation, observer-rated sedation, or DSST score were greater in females^8,13^. We also analyzed the PK-PD relationship using zolpidem concentration and changes in PD parameters from baseline. Only the slopes of the DSST score regression line were different between males and females, and females had less steep slopes, which means that males showed greater DSST score changes at the same concentration. Considering that the PK-PD relationship of zolpidem was similar in males and females, the difference in PD parameters seems to be owing to differences in zolpidem concentration.

The US Food and Drug Administration required the sponsor to change the labeling information of zolpidem owing to concerns that females have higher morning zolpidem levels than males, which leads to impaired driving ability^8^. A zolpidem level > 50 ng mL^−1^ is thought to be the cutoff value for the risk of driving impairment^9^. Two (one female, one male) subjects had zolpidem levels over 50 ng mL^−1^ at 8 h post-dose in our study. Even though zolpidem exposure was about 20–30% higher in females, it was not statistically significant. Reducing the dose to half of that prescribed for males in females might be insufficient to achieve the appropriate effect of zolpidem considering our results and that of previous studies and the lack of efficacy study results for the 5-mg dose of zolpidem.

Our study has several limitations. A placebo-controlled design is recommended for PD evaluations due to the placebo effect. However, it was difficult to obtain a placebo of zolpidem produced in accordance with local regulation and the study was conducted with an open-label design inevitably. While the PD tests we used are widely used in this field, there are some limitations. The sensitivity of DSST could be compromised because the motivation to complete the test could overcome mild sedation^38^ and VAS could be subjective and it is not appropriate to compare across a group of individuals at one time point. Even though the subject number of 30 was similar to previous studies of zolpidem, it was somewhat insufficient considering large inter-individual variability in PK and PD parameters. We used 4β-OH cholesterol level as an endogenous biomarker of CYP3A4 activity. Some studies argue that 4β-OH cholesterol does not represent the basal CYP3A activity^39,40^. A better biomarker of CYP3A4 metabolic activity should be explored in a future study.

In conclusion, the exposure of zolpidem was 30% increased in females and the VAS score profile was different between sexes. We suggest that the metabolic clearance mediated by CYP3A might not be the main cause of different exposure to zolpidem in both sexes because CYP3A activity was found to be higher in females than in males and the elimination t_1/2_ was similar. Lower body weight might be a more plausible explanation for the higher exposure in females. In addition, as the exposure level in females was much less than two-fold that in males, reducing the dose by half needs to be based on more concrete evidence.

## Supplementary Information

Supplementary Information.

## Data Availability

The datasets generated during and/or analyzed during the current study are available from the corresponding author on reasonable request.
