# Supplementary material for: Effect of CYP3A4 metabolism on sex differences in the pharmacokinetics and pharmacodynamics of zolpidem
Source: Sci Rep. 2021 Sep 27;11:19150. doi: 10.1038/s41598-021-98689-z (PMC8476623; doi:10.1038/s41598-021-98689-z)
Supplement: Supplementary file 1 — Supplementary Information. [file 41598_2021_98689_MOESM1_ESM.pdf]

Supplementary Table 1. Design information of studies that analyzed pharmacokinetics and/or pharmacodynamics of zolpidem

Reference No.	Number of subjects		Body weight (kg)[a]		IP	Route of administration	Parameters analyzed (PK and/or PD)
	Male	Female	Male	Female			
8	38	32	80	65	10 mg IR, 12.5 mg MR	PO	PK, PD
13	10	8	75.3 (10.5)	66.8 (8.7)	10 mg IR	PO	PK, PD
14	21 (19)[b]	15 (14)[b]	81.5 (11.2)	67.8 (9.7)	3.5 mg ZST, 10 mg IR	SL, PO	PK
15	13	11	80.3 (9.5)	67.4 (7.8)	1.0, 1.75, 3.5 mg ZST	SL	PK, PD
16	8	16	76.4 (5.9)	66.3 (6.6)	5 mg IR	PO	PK
17	25	25	NA	NA	10 mg IR	PO	PK
18	15	9	92 (10)	73 (11)	1.75, 3.5 mg ZST	SL	PK

[a] All values are presented as the arithmetic mean (standard deviation).

[b] The number of subjects included in the pharmacokinetic analysis are shown in parentheses.

Abbreviations: IP, investigational products; IR, immediate release; MR modified release; PD, pharmacodynamics; PK, pharmacokinetics; PO, per os (oral administration); SL, sublingual administration; ZST, zolpidem sublingual tablet

Supplementary Table 2. Summary of pharmacokinetic parameters from reference articles

Reference No.	IP	C_{max} (ng mL^{-1})		AUC_{last} (ng h mL^{-1})		AUC_{inf} (ng h mL^{-1})	
		Male	Female	Male	Female	Male	Female
8[a]	10 mg IR	133	148	NA	NA	518	680
13	10 mg IR	119 (40)	140 (43)	NA	NA	NA	NA
14[a]	10 mg IR	124 (37)	177 (53)	NA	NA	533 (312)	740 (185)
15[a]	3.5 mg ZST	53 (14)	77 (24)	187 (65)	280 (96)	198 (72)	296 (106)
16	5 mg IR	40 (16)	60 (19)	NA	NA	110 (68)	249 (133)
17	10 mg IR	169.11 (70.2)	217.83 (71.27)	507.86 (246.34)	784.20 (240.36)	524.78 (260.03)	831.63 (266.67)
18[a]	3.5 mg ZST (non-elderly)	60 (3.5)	65. (6.4)	238 (22)	250 (44)	260 (27)	268 (51)

(continued)

Reference No.	T_{max} (h)		CL/F (mL min^{-1})		CL/F (weight normalized) (mL min^{-1} kg^{-1})		$t_{1/2}$ (h)		PK parameter with statistically significant sex difference
	Male	Female	Male	Female	Male	Female	Male	Female	
8[a]	NA	NA	305	241	4	3.7	2.6	2.9	AUC_{inf}, CL/F
13	1.2 (0.35)	1.56 (1.18)	486 (260)	230 (60)	6.7 (4.3)	3.5 (0.9)	1.64 (0.42)	2.65 (0.97)	CL/F, $t_{1/2}$
14[a]	0.5 (0.7)	0.8 (1.8)	316 (153)	193 (54)	3.91 (2.12)	2.88 (0.87)	2.3 (0.9)	2.4 (0.5)	no *p* values presented
15[a]	0.6 (0.2)	0.7 (0.2)	307 (250)	179 (66)	4.0 (3.7)	2.7 (1.0)	2.4 (0.6)	2.5 (0.6)	C_{max}/dose, AUC_{last}/dose AUC_{inf}/dose, CL/F
16	0.8 (0.3)	1.2 (0.4)	820 (445)	376 (271)	11.0 (6.4)	5.8 (4.8)	1.5 (0.5)	2.4 (0.9)	no *p* values presented

17	0.94 (0.34)	0.79 (0.28)	458.5 (411.5)[b]	226.3 (96.3)[b]	NA	NA	2.02 (0.60)	2.63 (0.70)	AUC_{last}, AUC_{inf}, CL/F
18[a]	0.67	0.67	206 (20)	225 (36)	2.28 (0.25)	3.25 (0.62)	2.8 (0.2)	2.3 (0.3)	no significant difference

[a] Pharmacokinetic parameters of one dosage regimen are presented in this table for the studies that used several dosage regimens.

[b] The numbers are recalculated to match the unit (the unit was L h^{-1} in the original article).

Note: All values are presented as arithmetic mean (standard deviation) or arithmetic mean only for the parameters presented with other formats such as range or CV% in the original articles.

Abbreviations: C_{max}, maximum plasma concentration; T_{max}, time to reach C_{max}; AUC_{last}, area under the concentration curve from 0 to last measurable time; AUC_{inf}, area under the concentration curve from 0 to infinity; CL/F, apparent clearance; $t_{1/2}$, half-life; IR, immediate release; PK, pharmacokinetic; NA, not available; ZST, zolpidem sublingual tablet
